# Comparison of Healing Effect of Aloe Vera Extract and Silver Sulfadiazine in Burn Injuries in Experimental Rat Model

**Published:** 2014-01

**Authors:** Mohammad Reza Akhoondinasab, Motahhare Akhoondinasab, Mohsen Saberi

**Affiliations:** 1 Faculty of Plastic and Reconstructive Surgery, Burn Research Center, Iran University of Medical Sciences, Tehran, Iran;; 2 Faculty of Pharmacy, Tehran University of Medical Sciences, Tehran, Iran;; 3 Medicine, Quran and Hadith Research Center, Department of Community Medicine, Faculty of Medicine, Baqiyatallah University of Medical Sciences, Tehran, Iran

**Keywords:** Aloe vera, Silver sulfadiazine, Burn, Rat

## Abstract

**BACKGROUND:**

Wound healing is widely discussed in the medical literature. This study compared the healing effect of aloe vera extract and silver sulfadiazine in burn injuries in experimental rat model.

**METHODS:**

Sixteen rats were randomly assigned to one of two groups, each group 8 rats. A deep second-degree burn on the lower back and 3^rd^ degree burn on upper back of each rat were created with a standard burning procedure. Burns were dressed daily with aloe vera extract in group 2 and silver sulfadiazine in group 1. Response to treatment was assessed by digital photography during treatment until day 32. Histological parameters (PMN, epithelialization, fibrosis and angiogenesis) were assessed after biopsy of scar at the end of research.

**RESULTS:**

Wound healing was more visible in aloe vera group. Also the speed of healing in aloe vera group was better than silver sulfadiazine group.

**CONCLUSIONS:**

Based on our findings, aloe vera can be a therapy of choice for burn injuries.

## INTRODUCTION

Wound healing is widely discussed in the medical literature. Much research has been carried out to develop more sophisticated dressing that expedite the healing process and diminish the bacterial burden in wounds.^1^ Traditional forms of medicine especially herbal products deployed for centuries in Africa and Asia are under scientific investigation for their attributes in treatment of wound. Avicenna, the Persian physician and scholar (980-1037 AD) recommended medicinal plants, for dressing of wound in his famous book, Canon of medicine.^1^

Red Ginseng root extracts have also been used clinically as topical treatments for atopic suppurative dermatitis, wounds and skin inflammation.^2^ Herbal products seem to possess moderate efficacy with no or less toxicity and are less expensive as compared with synthetic drugs.^3^ There are several reports on using herbal drugs in healing of burn injuries.^4^^-^^7^ The kiwifruit originated >700 years ago in China. It was later introduced in New Zealand and California, where the first major planting occurred in 1960. 

Some clinical effects of kiwi fruit ingredients such as ascorbic acid (as a scavenger), antibacterial agents, and actinidin (a potent protein-dissolving enzyme) have been reported in the literature.^8^ Burn wound healing is one of major indications of aloe vera gel use in many countries.^9^^,^^10^ Clinical data on the treatment of psoriasis and Lichen ruber planus have confirmed long lasting ameliorative effects of BAC-3 (existing with high concentration in dirhamnolipid) when compared to conventional therapy using corticosteroids.^11^

For many years the effect of herbal medicine on burn wound has been noted. Herbal products seem to possess moderate efficacy with no or less toxicity and are less expensive as compared with synthetic drugs. Many plants and plants-derived products have been shown to possess potent wound-healing activity.^12^ *Spathodea campanulata Beauv.* (Bignoniaceae) is widely distributed through Africa and found in particular in Cameroon and Senegal. It is used in traditional herbal medicine for the treatment of ulcers, filaria, gonorrhea, diarrhea and fever. *S. campanulata* was also known in Cameroon traditional medicine to have a healing activity in burn wounds.^8^

Combudoron, composed of extracts from arnica and stinging nettle is used for the treatment of partial thickness burns and insect bites in Europe. Nettle root extracts contain at least 18 phenolic compounds and 8 lignans.^13^ Healing of burn is still a challenge in modern medicine and there are a few drugs capable of accelerating wound healing. As alternative plants are rich sources to survey.^14^

Traditionally, fresh leaves or decoction of* Chromolaena odorata* have been used throughout Vietnam for many years as well as in other tropical countries for the treatment of leech bite, soft tissue wounds, burn wounds, skin infection and dento-alveolitis.^15^ Combudoron also seems to have positive effects on healing of grade 2 laser induced burns which deserve further investigation.^16^

Swift eschar separation with a resulting wound-bed that appeared pink and viable suggests that kiwifruit may help in the management of patients with deep burns.^17^^,^^18^ This study compared the healing effect of aloe vera extract and silver sulfadiazine in burn injuries in experimental rat model.

## MATERIALS AND METHODS

**Fig. 1:**
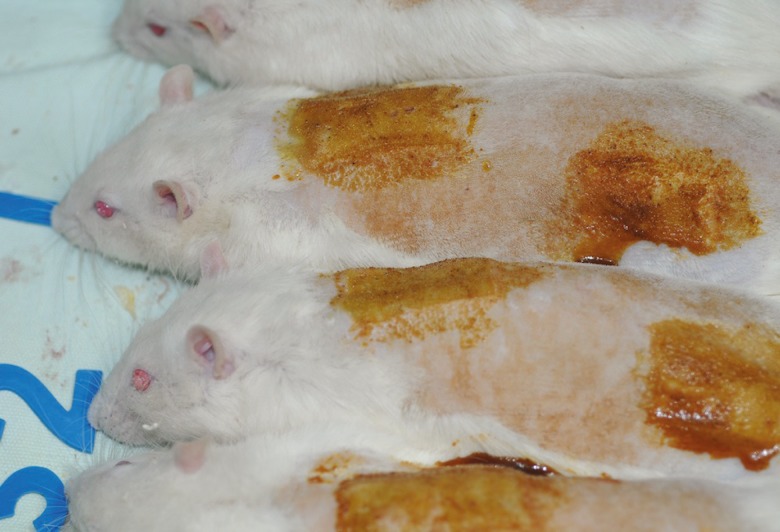
3^rd^ degree burn over upper back and 2^nd^ degree burn over lower back in 2^nd^ session

In a randomized clinical trial, 16 Wistar-albino male rats (average weight: 300-350 gr, average age: 3-4 months) were randomly divided into 2 equal groups (1: topical silver sulfadiazine treated group, 2: topical aloe vera group). They were all in a sheltered environment (temperature: 20-25°C; humidity: 65-75%) under the supervision of a veterinarian. During experimentation, the rats were fed with usual rat chow and tap water and each rat was kept in a separate cage. All rats were handled according to the ethical principles for animal experiments of the international council for animal protection. All experimental procedures were agreed by the research ethics committee of the university. Rats were anesthetized with inhalational anesthesia using xylazine (10 mg/kg) and ketamine hydrochloride injection (50-100 mg/kg intramuscularly) to increase the depth of anesthesia. The skin on the dorsum was shaved with an electrical clipper. A deep, second-degree burn wound was created with a hot plate (diameter: 4×2 cm) at an identical temperature (warmed 5 minutes within boiling water and placed for 10 seconds on the skin with an equal pressure) over lower back and a third degree burn over upper back by 30 seconds of pressure (Figure 1).^1^ Then surface of wounds were covered by corresponding ointment and no dressing were applied. These ointments were used daily. For assessment of wound healing, digital photography was taken every 4 days under general anesthesia. The photographs then are assessed by software Image j and the percentage of healing was determined. Histologic parameters (PMN, epithelialization, fibrosis and angiogenesis) were assessed on biopsy specimens of wounds at the end of the study. Every specimen was provided under general anesthesia by resection of healed area and surrounding normal skin. Histological criteria was defined as following: for fibrosis (collagen bundles): normal bundle: 2, disorganized/edematous: 1, and amorphous: 0. For PMN, 40x field: 0-10: 2, 11-40: 1, >40: 0. Angiogenesis in 3 degree: mild, moderate and severe. Epithelialization was expressed as positive and negative.

## RESULTS

**Fig. 2:**
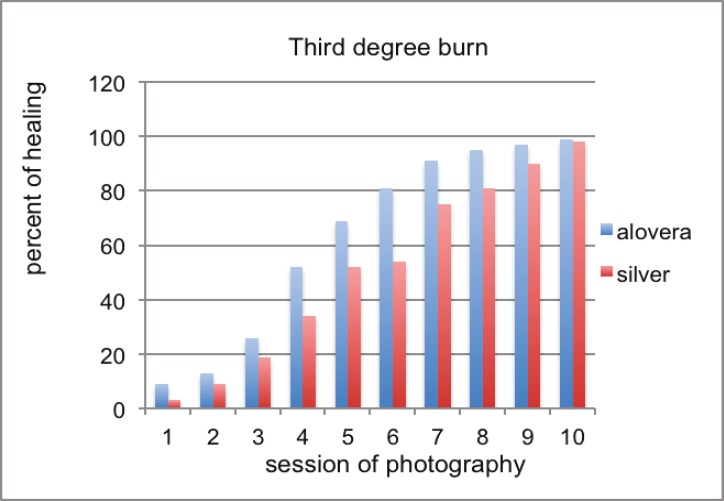
Comparison of healing of third-degree burns

**Fig. 3:**
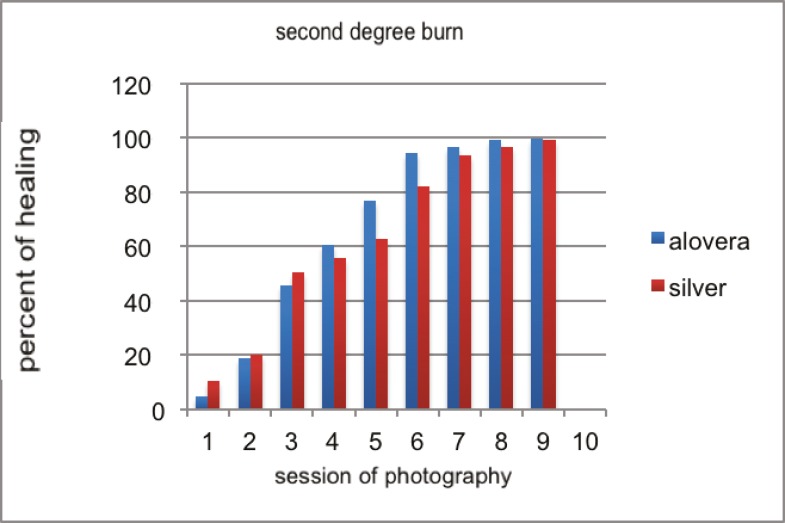
Comparison of healing in second degree burns

This was an experimental study using male Sprague–Dawley rats. We investigated the healing properties of aloe vera leaf extract. One of the animals died in silver sulfadiazine group. In 3^rd^ degree burns, wound healing was significantly more in aloe vera group (Figure 2 and 3), but for second degree burns, the difference was not as significant as third-degree burns. Pathological assessments of specimens encompassed fibrosis, angiogenesis, inflammation and epithelialization. Epithelialization was more evident in aloe vera group. In second-degree wounds except in 2^nd^, 8^th^ and 11^th^ sessions, the difference between groups was significant (P<0.005) and the best results belonged to aloe vera group. In third degree burns, except in 2^nd^, 4^th^ and 11^th^ sessions, the difference between groups was significant (P<0.005) and aloe vera had more healing effect.

## DISCUSSION

The skin is one of the largest organs in the body that performs numerous vital functions including fluid homeostasis, thermoregulation, immunologic, neurosensory and metabolic functions. The skin also provides primary protection against infection by acting as a physical barrier.^8^ When this barrier is damaged, pathogens have a direct route to infiltrate the body, potentially resulting in infection. The sequence of events that repairs the damage is categorized into three overlapping phases: inflammation, proliferation and tissue remodeling. The normal healing process can be impeded at any step along its path by a variety of factors that can contribute to impaired healing. Impaired wound healing may be a consequence of pathologic states associated with diabetes, immune disorders, ischemia, venous stasis and injuries such as burn, frostbite and gunshot wounds.^8^ The final step of the proliferative phase is the epithelialization. It involves migration, proliferation and differentiation of epithelial cells from the wound edges to resurface the defect. In open full thickness burn wounds, epithelialization is delayed until a bed of granulation tissue is established to allow migration of epithelial cells.^12^

Several studies showed that burn infection is the main cause of mortality in patients with extensive burns. Therefore, many researchers tried to achieve appropriate treatment methods to reduce the risk of wound infections and to shorten the period of treatment of patients with burn wounds.^1^ Some of these treatments involve using topical antimicrobial agents which effectively reduce mortality rate of burns.^4^^-^7 One of these antimicrobial topical ointment is 1% silver sulfadiazine, with advantages such as easy and convenient use, not to create pain when consumed, yielding low toxicity and sensitivity and having anti-bacterial effect, which made it known as the gold standard of anti-microbial topical drugs for patients with burns and turned it to the main consumed medicine in treatment of burn wounds around the world.^3^^,^^9^ Burn management entails significant duration of hospital stay, expensive medication, multiple operative procedures and prolonged period of rehabilitation. Topical anti-bacterial agents and disinfectants are good in protecting against infection, but the occurrence of allergic reactions and skin irritations to these agents reduces the rate of skin regeneration and increases the recovery time.^8^

The ultimate burn dressing wound is inexpensive and comfortable and it would not only allow the burn to heal rapidly, but also clean the wound and debride fragments of separated eschar and devitalized tissue and have antibacterial activity. A wide variety of substances have been reported to be useful in the treatment of burn wound.^4^^-^^9^ Healing of burn is still a challenge in modern medicine and there are a few drugs capable of accelerating wound healing and as an alternative plants were rich sources to survey.^4^^-^^7^^,^^15^ For many years, the effect of herbal medicine on burn wound has been noted. Herbal products seem to possess moderate efficacy with no or less toxicity and are less expensive as compared with synthetic drugs. Many plants and plants-derived products have been shown to possess potent wound-healing activity.^4^^-^^8^ Eupolin ointment, a formulation prepared from the aqueous extract of the leaves of *C*. *odorata *(formerly *Eupatorium odoratum*) has been licensed for clinical use in Vietnam.^17^ Most of the medicines are mixture of several plants, but none of these traditional ointments were scientifically studied. In our study, aloe vera extract was compared with silver sulfadiazine as the standard treatment for burn wounds in rat. The actual mechanism of improved healing is still unclear. The probable mechanism are providing necessary material for healing, increasing blood flow to burn area, decreased inflammatory response, and decreasing rate of infection. The healing time in grade 3 burns, in aloe vera group was significantly shorter than silver sulfadiazine group. This effect might be due to major role of wound contraction in third degree burn wounds in skin of rats. Wound healing in rat skin does not perfectly mimic human skin wound healing because the skin morphology is different (rats are described as loose-skinned animals) and ‘‘loose’’ skin allows wound contraction to play a significant role in closing rat skin wounds. Consequently, wound contraction is usually more rapid than epithelialization.^12^ Human has tight skin, and this difference makes the comparison with loose-skinned animals more difficult. Although there are inherent drawbacks in using rats for comparisons with human skin wound healing, there are also advantages in the use of rats as a research model, such as the availability of a broad knowledge based on rat wound healing gained from years for previous research.^12^

Aloe vera (Aloe vera Linn, synonym: aloe vera barbadensis Mill) is in family Liliaceae, which is a tropical plant easily grown in hot and dry climates including Thailand. Numerous cosmetics and medicinal products are made from the mucilaginous tissue, called aloe vera gel, located in the center of the aloe vera leaf. Aloe vera gel has been used for many indications since the Roman era or even long before. Burn wound healing is one of major indications of aloe vera gel use in many countries.^10^ A recent review of four clinical trials investigating the effect of Aloe vera on burn wounds found that aloe vera significantly shortened the wound healing time (by approximately eight days) compared to control. They concluded that it may be an effective treatment for first and second degree burns.^14^ The results of the study provided permission for a start in human study. We hope a new burn ointment can be introduced by usage of herbal medicines with less adverse effects and shorten the period of healing thus decrease the rate of hypertrophic scar. Our findings denotes to recommendation of aloe vera in healing of burn injuries as an inexpensive and available herbal medicine.

## Footenote

Please cite this paper as:

Akhoondinasab MR, Akhoondinasab M, Saberi M. Comparison of Healing Effect of Aloe Vera Extract and Silver Sulfadiazine in Burn Injuries in Experimental Rat Model. World J Plast Surg 2014;3(1):29-34.
